# Intergenerational Transmissions of Mother–Adolescent Smartphone Dependency: The Mediating Role of Negative Parenting and the Moderating Role of Gender

**DOI:** 10.3390/ijerph17165871

**Published:** 2020-08-13

**Authors:** Chanhee Kim, Kyung Im Kang, Nayoon Lee

**Affiliations:** 1 College of Nursing, Dong-A University, Busan 49201, Korea; chany131@dau.ac.kr; 2 Department of Nursing, College of Medicine, Dongguk University, Gyeongju 38066, Korea; 3 College of Nursing, Catholic University of Pusan, Busan 46252, Korea; nayoon@cup.ac.kr

**Keywords:** smartphone dependency, intergenerational transmission, negative parenting, gender

## Abstract

Given the prevalence and undesirable consequences of smartphone dependency among adolescents, it is necessary to explore the influencing factors of adolescent smartphone dependency. The aim of this study was to examine the intergenerational transmission of mother–adolescent smartphone dependency and the mediating role of negative parenting, moderated by adolescent gender. Data for 2541 middle school students (mean aged = 13 years)–mother dyads were obtained from the first wave of the Korean Children and Youth Panel Survey 2018 (KCYPS 2018). The moderated mediation model using Hayes PROCESS macro (Model 14) was employed to test the study hypothesis. The moderated mediation model revealed that maternal smartphone dependency was associated with adolescent smartphone dependency. Perceived negative parenting mediated this link and adolescent gender moderated the relationship between negative parenting and adolescent smartphone dependency, especially for adolescent girls. Our findings showed that both maternal smartphone dependency and negative parenting were determinants of adolescent smartphone dependency, suggesting that both factors were important for understanding these issues. Moreover, the mediating role of negative parenting (between maternal and adolescent smartphone dependency) implies that parental education programs designed to improve negative parenting may reduce adolescent smartphone dependency, especially for adolescent girls.

## 1. Introduction

Smartphones, which allow users to quickly and easily obtain information, have become essential devices in daily life. South Korea has the highest smartphone usage rate in the world with 95% of the total population using smartphones [1]. Despite the many advantages of using these devices, many studies have reported on the negative effects of their use. The most prevalent problem is smartphone dependency (characterized by daily life disturbances, virtual world orientation, withdrawal, and tolerance), which can lead to harmful consequences [2]. In a non-clinical population, the degree of dependency was related to well-being, but the effect was small, therefore there may be concerns about over-pathologizing smartphone dependency [3]. However, considering the statistics on smartphone use in South Korea, it would be beneficial to pay attention to smartphone dependency. According to data from a smartphone over-dependency survey conducted in 2019, the proportion of those in the over-dependency risk group (high-risk and potential-risk groups) in South Korea was 20.0%, and the age group with the largest proportion of those in the over-dependency risk group was adolescents, with 34.7% of middle school students being the most vulnerable to smartphone dependency [2]. Smartphone dependency is associated with other mental health problems such as depression and anxiety [4,5]. If smartphone dependency and associated mental health problems occur in early adolescence, symptoms remain and worsen until late adolescence [5]. Adolescents who are highly dependent on smartphones tend to be socially isolated and are at increased risk of emotional and behavioral problems that can lead to adverse effects on their health, learning, and development [6,7]. Considering the prevalence and negative effects of smartphone dependency in adolescents, it is necessary to identify its related factors and underlying mechanisms. This will provide a better understanding of smartphone dependency in adolescents and aid in the development of preventive interventions.

Factors influencing smartphone dependency in adolescents include personal, parental, and environmental factors [8,9]. In particular, the prevalence of smartphone dependency in adolescents with parents who also have the problem is much higher compared with adolescents with parents who do not have the problem [2].

Goodman and Gotlib’s intergenerational transmission model [10] provides better theoretical understanding of the relationship between maternal and adolescent smartphone dependency. The model suggests that children’s psychological problems, including depressive symptoms and anxiety, are intergenerationally transmitted through a variety of mechanisms, such as the transmission of parents’ emotional problems, maladjusted behavior, and stress in family relationships. In particular, social anxiety that appeared to be strongly related to phubbing could be transmitted from the mother to her children [11,12]. In addition, subsequent studies have revealed that behavioral addiction can also be intergenerationally transmitted [13,14]. These ideas suggest that smartphone dependency can be intergenerationally transmitted from mother to adolescent.

Social learning theory, developed by Bandura [15], can help to explain intergenerational transmission. The theory suggests that behavior is learned by observing and imitating others. Based on social learning theory, when parents frequently use smartphones, their children observe and unconsciously imitate this behavior. Parental smartphone dependency can be transmitted to their children through various mechanisms. In particular, the smartphone dependency of mothers, who generally spend the most time with their children, can have a great impact on their children [16].

The empirical evidence has shown that parents’ smartphone dependency is linked to children’s smartphone dependency [2,16,17]. However, studies investigating how and when the parent’s, especially the mother’s, smartphone dependency relates to the children’s smartphone dependency remain scarce. Thus, it would be worthwhile to examine the intergenerational transmission of smartphone dependency from mother to adolescent and the underlying mechanism of mediation and moderation of this link. This will provide better understanding for the development of preventive interventions for smartphone dependency in adolescents.

### 1.1. The Mediating Role of Negative Parenting Style

Studies have reported that maternal smartphone dependency has negative effects on parenting. For instance, mothers may feel guilty for not doing their best to raise their children, which can negatively affect their sense of parenting efficacy [18,19]. Mothers who are dependent on smartphones are overly immersed in smartphone use and do not respond sensitively to their children’s needs [20,21], which deteriorates the quality of mother–child communication and interaction [20]. This interferes with the formation of healthy attachment relationships between mothers and their children [22] and ultimately leads to negative parenting characterized by rejection, chaos, and coercion [23]. The nature of negative parenting, in turn, leads to weakening in the relationship and interference with the competency and development of psychological autonomy in children [23].

Above all, negative parenting style affects children’s smartphone dependency. Family environment factors also act as important predictors of children’s smartphone dependency [24,25]. In particular, a negative parenting style including rejection or coercion directly increased children’s smartphone dependency [6,26,27]. Moreover, a negative parenting attitude indirectly affected children’s smartphone dependency through depression, aggression [28], low quality of communication [29], neuroticism, and psychological maltreatment [27]. Therefore, it is necessary to examine the mediating effects of negative parenting on the relationship between maternal smartphone dependency and children’s smartphone dependency.

### 1.2. The Moderating Role of Child Gender

There are gender differences in the patterns of internet and smartphone use in adolescents. Adolescent boys are more vulnerable to internet addiction, whereas adolescent girls are more vulnerable to smartphone dependency [30,31,32]. There are also gender differences in smartphone use purposes in this age group. Adolescents boys use smartphones mainly for playing video games, whereas adolescents girls use smartphones for various tasks, such as texting, taking photos, listening to music, and watching videos [8]. Such gender differences in the purpose of using smartphones in adolescents may be because girls have more relationship-oriented characteristics, compared to boys. The characteristics of adolescent girls can make them more sensitive to parents’ parenting styles. In particular, women tend to focus more on the negative aspects [33,34], making them more vulnerable to negative relationships, namely, negative parenting [35,36]. In addition, being an adolescent girl may moderate the effects of negative parenting style on adolescent smartphone dependency more than being an adolescent boy. However, few studies have examined the moderating effects of gender on the relationship between negative parenting and adolescent smartphone dependency.

**Figure 1:**
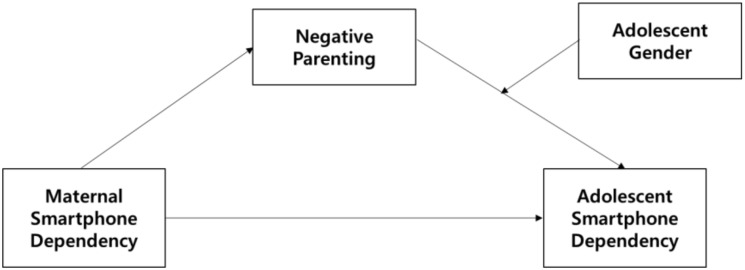
Conceptual framework of the current study.

This study aimed to examine the intergenerational transmission of mother-child smartphone dependency and the moderated and mediating role of negative parenting and adolescent gender. The conceptual model of this study is presented in Figure 1. The proposed hypotheses were as follows:

**Hypothesis** **1** **(H1).**  *Maternal smartphone dependency would be positively associated with adolescent smartphone dependency*.

**Hypothesis** **2** **(H2).**  *Negative parenting would mediate mother–adolescent smartphone dependency*.

**Hypothesis** **3** **(H3).**  *Adolescent gender would moderate the indirect association between negative parenting and adolescent smartphone dependency*.

## 2. Materials and Methods

### 2.1. Study Participants and Procedure

The data used in this study were obtained from the first wave of the Korean Children and Youth Panel Survey 2018 (KCYPS 2018), an ongoing, nationally representative, longitudinal study conducted by the National Youth Policy Institute. Data from KCYPS have been accumulated yearly from nationally representative panels of elementary and middle school students since 2018. The Korean Children and Youth Panel Survey 2018 was carried out using a stratified multistage clustering sampling method. In the present study, we used the data on first-year middle school student (mean age = 13 years) and mother dyads, collected in 2018 (Wave1). Of the 2590 potential participants, data for 2541 students who were using smartphones and their mothers were used. 

### 2.2. Measures

In this study, data were collected on mother and adolescent smartphone dependency, and perceived negative parenting. Covariates included sociodemographic characteristics such as perceived economic status, mother’s education, and adolescent depressive symptoms and level of self-esteem.

#### 2.2.1. Mother–Adolescent Smartphone Dependency

To assess mother and adolescent smartphone dependency, the Smartphone Addiction Proneness Scale (K-SAS) developed by the National Information Society Agency in Korea [37] was used. The K-SAS has been validated for the screening of smartphone addiction in adults and adolescents [37]. This scale consists of 15 items and 4 domains: daily life disturbance, virtual world orientation, withdrawal, and tolerance. The responses were rated on a four-point Likert scale ranging from 1 = ‘not at all’ to 4 = ‘very strongly’. The positive items are reverse-coded, and higher scores indicate that the mother and middle school student have higher levels of smartphone dependency. Cronbach’s alpha for the mother and child smartphone dependency were 0.76 and 0.80 in this study.

#### 2.2.2. Negative Parenting

Perceived negative parenting by adolescents was measured using the Korean Version of the Parents as Social Context Questionnaire for Adolescents (K-PSCQ) [38], which was originally developed by Skinner, Johnson, and Snyder [39]. The scale assesses six parental dimensions: warmth, rejection, structure, chaos, autonomy support, and coercion. We examined the three negative dimensions: rejection, coercion, and chaos. Responses were based on a four-point Likert scale, and higher scores indicate higher levels of negative parenting. Cronbach’s alpha for the negative parenting was 0.87 this this study.

#### 2.2.3. Covariates

In this study, sociodemographic characteristics such as perceived economic status and mother’s education were included as covariates. Perceived economic status and mother’s education were assessed using the mothers’ reports. Adolescent gender was used as a moderator. Adolescent depressive symptoms and self-esteem, which were found to be important factors of adolescent smartphone dependency in previous studies [4,29,40] were also controlled as covariates. Perceived economic status was coded as 0 = low, 1 = average, 2 = high. Mother’s education was coded as 0 = equal to or below high school, 1 = two-year college graduate, 2 = four-year college graduate or above. The Symptom Checklist-90-Revised [41] was used to assess adolescent depressive symptoms. This scale consists of 10 items on a four-point Likert scale, ranging from 1 = ’not at all’ to 4 = ’very strongly’. A higher score indicates a higher level of depression. Cronbach’s alpha for the depression was 0.92 in this study. The Rosenberg Self Esteem Scale [42] was used to assess adolescent self-esteem. This scale consists of 10 items with responses based on a four-point Likert scale, ranging from 1 = ’very true of me’ to 4 = ‘not at all true of me’. The negative items were reverse-coded, and higher scores indicate higher levels of self-esteem. Cronbach’s alpha was 0.87 in this study.

### 2.3. Data Analysis

Data analysis was conducted using SPSS version 22.0 (IBM Corp., Armonk, NY, USA). First, to handle missing data, full information maximum likelihood (FIML) was used. Skewness and kurtosis for each variable were checked to determine whether the data had a normal distribution. Then, descriptive statistics and bivariate correlations analyses were conducted. Next, to test the study hypotheses, the moderated mediation model was employed using Hayes PROCESS macro (Model 14) [43]. This moderated mediation model yields multiple coefficients of direct and indirect effects. Adolescent smartphone dependency was entered into the moderated mediation model as the outcome variable, mother smartphone dependency as the predictor variable, negative parenting as the mediator, and adolescent gender as a moderator of the relationship between negative parenting and adolescent smartphone dependency. The 95% bias corrected confidence interval from 5000 resamples was generated using the bias-corrected bootstrapping method. The number of bootstrapping size was 5000. The predictor and moderator variables were mean centered. Significant indirect effects were identified when the confidence interval (CI) did not include zero.

## 3. Results

### 3.1. Demographic Characteristics and Correlations among the Study Variables

Of the 2541 participants, 54.1% (1375) were boys, and the mean age was 13 years (standard deviation [SD] = 0.13). Most participants’ (76.3%) perceived their economic status as average level. Over half of the mothers (59.8%) had at least a 2-year college level of education.

As shown in Table 1, the mean scores of maternal and adolescent smartphone dependencies were 27.60 (SD = 5.84) and 31.60 (SD = 5.81), respectively. Perceived negative parenting had the mean score of 23.98 (SD = 6.29). For each variable, skewness ranged from −0.06 to 0.62 and kurtosis ranged from −0.19 to 0.37. Based on reference values of an absolute skewness value ≤ 2 or an absolute kurtosis ≤ 4, assumptions of normality for the variables were met. Self-esteem was negatively correlated with maternal smartphone dependency (r = −0.659, *p* < 0.001) and adolescent smartphone dependency (r = −0.406, *p* < 0.001). Maternal smartphone dependency was positively correlated with adolescent smartphone dependency (r = 0.166, *p* < 0.001). Negative parenting was positively correlated with maternal smartphone dependency (r = 0.154, *p* < 0.001) and adolescent smartphone dependency (r = 0.334, *p* <0.001).

### 3.2. Moderated Mediation Testing

**Figure 2:**
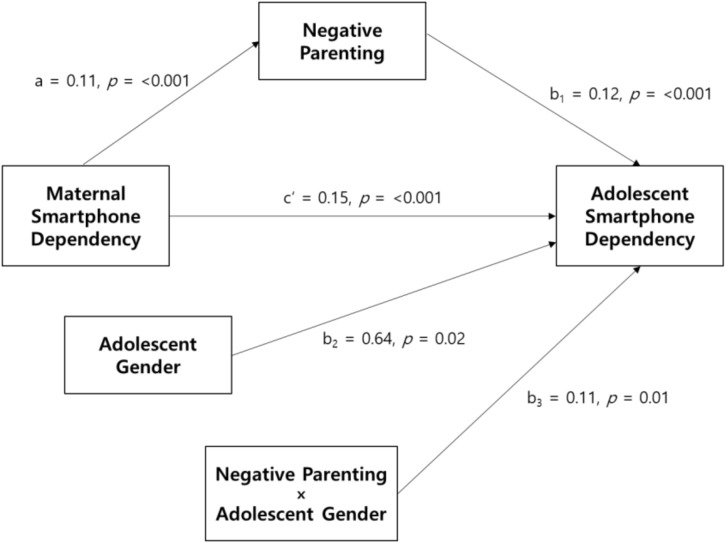
Unstandardized regression coefficient for the moderated mediation model.

**Figure 3:**
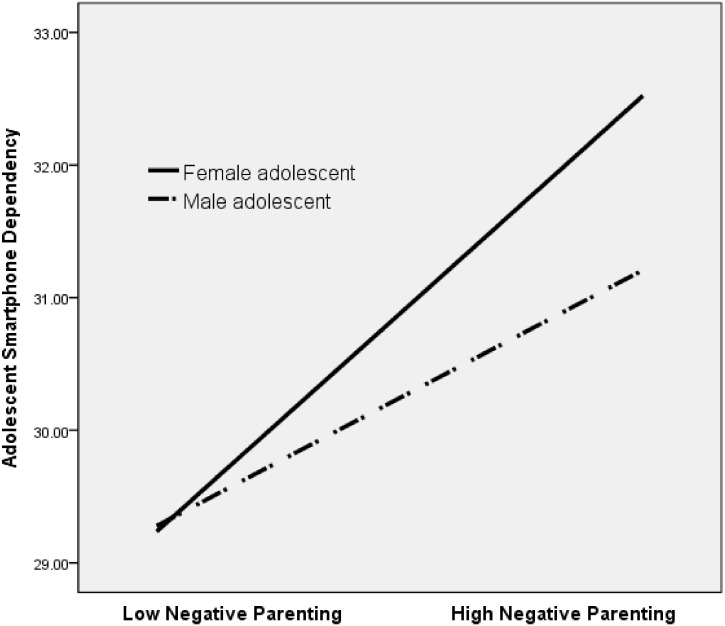
Moderation of the effect of negative parenting on adolescent smartphone dependency by gender.

Model 14 of PROCESS macro [37] was used to test the study hypothesis, that is, to examine the relationship between mother and adolescent smartphone dependency, and the mediating role of perceived negative parenting and moderating role of adolescent gender. In all analyses, we controlled for covariates including perceived economic status, mother’s education, and adolescent depressive symptoms and self-esteem. Figure 2 presents the unstandardized regression coefficients for each path in the moderated mediation model. As shown in Table 2, the direct effect of maternal smartphone dependency on adolescent smartphone dependency was significant (path c’: β = 0.12, *p* < 0.001, 95% CI 0.077, 0.155). In the mediation analysis, maternal smartphone dependency positively predicted perceived negative parenting (path a: β = 0.11, *p* < 0.001, 95% CI 0.073, 0.139), and perceived negative parenting positively predicted adolescent smartphone dependency (path b_1_: β = 0.12, *p* < 0.001, 95% CI 0.095, 0.211). The findings support Hypothesis 2. Next, the moderated mediation showed a significant interaction effect between negative parenting and adolescent gender on adolescent smartphone dependency (path b_3_: β = 0.11, *p* = 0.01, 95% CI 0.026, 0.190). Results of the analysis of the conditional indirect effect of maternal smartphone dependency on adolescent smartphone dependency through perceived negative parenting for adolescent boys and girls were as follows: As shown in Table 3, the indirect effect was significant for both boys (β = 0.02, standard error [SE] = 0.00, 95% CI 0.01, 0.03), and girls (β = 0.03, SE = 0.01, 95% CI 0.02, 0.40). Hypothesis 3 was supported. However, the magnitude of this relationship was stronger for females. Figure 3 shows the moderation effect of gender on the relationship between negative parenting and adolescent smartphone dependency. The index of moderated mediation was significant, which shows that the indirect effects in the model were significantly different for adolescent gender (index = 0.01, Boot SE = 0.01, 95% Boot CI 0.003, 0.022).

## 4. Discussion

Smartphone dependency is rapidly becoming a public health issue across the globe. Using a large nationally representative sample of Korean mother and adolescent dyads, we revealed that maternal smartphone dependency was associated with adolescent smartphone dependency. Such intergenerational transmission was mediated via negative parenting and this indirect relationship was moderated by adolescent gender. Specifically, maternal smartphone dependency can exacerbate perceived negative parenting (such as rejection, or coercion, chaos); potentially resulting in increased adolescent smartphone dependency. The magnitude of the exacerbating effect is greater for adolescent girls than adolescent boys.

Consistent with hypothesis H1, our findings revealed that maternal smartphone dependency was positively associated with adolescent smartphone dependency (H1). This is in line with the findings of a previous study that demonstrated that parent phubbing was positively associated with adolescent phubbing [44]. Moreover, parental internet use has been found to be significantly linked to adolescent internet use [45,46]. Studies on TV viewing [47], substance use [48,49], and gambling [13,14] also reported similar findings. As discussed in the introduction, this finding can be explained with Bandura’s social learning theory [15]. Social learning theory suggests that learning occurs by observing and imitating the behavior of others. Parents play a fundamental role in shaping adolescents’ behaviors. As role models, parents directly influence their child’s level of smartphone dependency through their own smartphone use [46]. When parents use their smartphones excessively, their children, even infants, are more likely to act like their parents [50]. Even though parents do not intentionally encourage their children to dysfunctionally use smartphones, children still learn and imitate the behaviors of their parents [51]. In addition, behavioral addiction problems can be transmitted intergenerationally as a result of continuous exposure to maladaptive parental behaviors or addiction-related psychopathology [13,14]. This suggests the possibility that maternal dependency on smartphones can be passed on to children, which adds to the evidence of intergenerational transmission of behavioral addiction problems from mother to adolescent. Thus, mothers should be encouraged to reduce their smartphone dependency to reduce smartphone dependency among adolescents.

This study revealed that maternal smartphone dependency can affect adolescent smartphone dependency through negative parenting (i.e., through rejective, coercive, and chaotic attitudes and behavior), supporting hypothesis H2. In other words, maternal smartphone dependency can aggravate perceived negative parenting and lead to increased adolescent smartphone dependency. Similarly, one previous study found that maternal smartphone addiction tendency had a negative influence on parenting behaviors of mothers with preschoolers [52]. Kildare and Middlemiss [20], conducted a literature review and found that parents who excessively used mobile phones are less sensitive and responsive to their children, and that this behavior leads to poor parent-child interaction. Maternal smartphone dependency was also found to increase parenting stress [53] and negative parenting attitudes [54]. This may be explained by findings from previous studies that indicate that higher levels of smartphone dependency can result in mental health problems such as depression, anxiety, and low self-esteem [55] that can negatively influence parenting behavior toward adolescent, and promote rejection, coercion and chaos.

This study also found that perceived negative parenting predicts adolescent smartphone dependency. This finding is consistent with those of previous studies that indicate that neglective or harsh parenting has negative effects on adolescent smartphone dependency [6,56,57]. Adolescents who experience negative parenting may develop smartphone dependency; adverse experiences lead to psychological distress, which can in turn increase excessive smartphone use. Moreover, smartphone use can serve as an avoidance strategy to divert negative emotional experiences. It has been found that distressed individuals often use smartphones to cope with their negative emotions [58]. Our findings suggest that negative parenting plays a significant mediating role in increasing adolescent smartphone dependency. Changing negative parenting behavior (i.e., rejection, coercion, and chaos) may prevent intergenerational transmission of smartphone dependency.

The findings also revealed a moderating role of gender in the association between negative parenting and adolescent smartphone dependency (H3). The results suggest that maternal smartphone dependency influences smartphone dependency among both adolescent boys and girls indirectly through negative parenting; however, the strength of the intensifying effect is significantly greater for girls than boys. The mediating role of negative parenting differed among boys and girls. Similar findings showed that parental control is more strongly associated with substance use in girls than in boys [59]. In addition, it has been observed that parent-child communication problems do not affect smartphone dependency in adolescent boys but affected smartphone dependency in adolescent girls [60]. Adolescent girls are likely to be more sensitive or attuned to parents’ negative responses [36] and thus they are more influenced by negative parental practices, which may increase the likelihood of smartphone dependency. Our findings regarding the moderating role of gender indicate the need for gender-specific intervention strategies to decrease smartphone addiction among adolescents.

Interestingly, in this study, self-esteem was the strongest predictor for both mother and adolescent smartphone dependency. Self-esteem is known to affect the smartphone dependency of young adults as well as negative parenting style of parents [26,61]. In addition, it has been reported as one of the direct and indirect psychological factors that influence adolescent smartphone dependency [28,29]. These results suggest that the programs for promoting self-esteem can help reduce negative parenting style and smartphone dependency for both parents and their children.

## 5. Limitations and Implications

There are several limitations that should be noted. First, the present study employed a cross-sectional study design, which limits interpretations of causality. Moreover, reverse causality in the relationship between negative parenting style and adolescent smartphone dependency cannot be excluded given the bidirectional nature of influence in the parent-child relationship [62] and the reciprocal relationship between parenting and internet use [63]. Thus, future research with longitudinal study designs could address issues of the causal relationships between the variables examined in this study. These studies may also identify accumulating effects of negative parenting on change in adolescent smartphone dependency. Furthermore, in our study, negative parenting was assessed by the adolescent, and even though we controlled for adolescent depression and self-esteem, these could have affected their responses. Discrepancies between adolescent and parent reports regarding parenting were found in previous studies [64,65]. Further studies assessing parenting based on parental reports are needed. Moreover, the effects of other confounding variables (i.e., self-control, relationship with friends, and academic achievement) on adolescent smartphone dependency were not controlled in this study and thus such variables should be controlled in future studies. Finally, although our findings were all statistically significant, the values of the effect sizes were small, therefore considerations should be taken when interpreting our results.

Despite these limitations, our results from a nationally representative study add knowledge to the current literature by examining the moderated mediation model, shedding light on the intergenerational transmission of mother-child smartphone dependency. Early adolescence is a period for shaping and modifying undesirable behaviors such as smartphone dependency. Parental mediation, specifically restrictive methods to regulate adolescent smartphone use, was found to be less effective, even harmful [66,67]. Our findings clearly indicate that mothers should reduce their own smartphone dependency in order to address their children’s smartphone dependency, offering an effective method. Efforts to reduce negative parenting (i.e., neglect, coercion, and chaos) can also diminish adolescent smartphone dependency, especially for girls. In previous studies, parenting training was found to have effects on externalizing problems of youth [68,69]. Thus, smartphone dependency among adolescents can be reduced through family-based approaches that focus on reducing negative parenting and maternal smartphone dependency. Based on the characteristics by the gender of the adolescents, education, activities, and counselling for parents, especially mothers, are required to guide desirable parenting and reduce smartphone dependency. It may also be important to provide various approaches to address the factors that affect negative parenting or maternal smartphone dependency (e.g., self-esteem) in preventing and reducing smartphone dependency among adolescents.

## 6. Conclusions

We are living in the smartphone era. We cannot deny the benefits of using our smartphones; however, smartphone dependency issues are rapidly becoming a major problem globally, especially among adolescents. Considering the increasing rates of smartphone dependency among youth and the harmful effects on adolescent health, learning, and development, our findings have significant implications, linking maternal smartphone dependency to adolescent smartphone dependency. Moreover, negative parenting may be an important mediating factor of intergenerational transmission of mother-child smartphone dependency, especially for adolescent girls. Our findings highlight the importance of training for parents to reduce negative parenting and maternal smartphone dependency.

## Figures and Tables

**Table 1:** Means, standard deviations and correlations for the variables by adolescent gender.

Variables	1	2	3	4	5
1. Depression	1	-	-	-	-
2. Self Esteem	0.174 **	1	-	-	-
3. Maternal Smartphone Dependency	−0.164 **	−0.659 **	1	-	-
4. Negative Parenting	0.004	0.089 **	0.154 **	1	-
5. Adolescent Smartphone Dependency	−0.042 *	−0.406 **	0.166 **	0.334 **	1
**Mean (SD)**	18.03 (6.39)	29.92 (5.03)	27.60 (5.84)	23.98 (6.29)	31.60 (5.81)
Range	10–40	11–40	15–60	12–48	15–60
Skewness	0.62	−0.21	0.36	0.36	−0.06
Kurtosis	0.03	−0.19	−0.13	0.09	0.37

SD = standard deviation * *p* < 0.01, ** *p* < 0.001.

**Table 2:** Moderated mediation testing results

Outcome	Predictors	Path	b	SE	*p*	LLCI	ULCI
Negative parenting	Constant	-	4.01	1.38	0.004	1.31	6.70
	Maternal Smartphone dependency	a	0.11	0.02	<0.001	0.073	0.139
	R^2^ = 0.23, F = 146.45, *p* < 0.01	-	-	-	-	-	-
Adolescent smartphone dependency	Constant	-	33.69	1.62	<0.001	30.52	36.86
	Maternal Smartphone dependency	c’	0.12	0.02	<0.001	0.077	0.155
	Negative parenting	b_1_	0.15	0.03	<0.001	0.095	0.211
	Gender	b_2_	0.64	0.27	0.02	0.106	1.164
	Negative parenting × gender	b_3_	0.11	0.04	0.01	0.026	0.190
	R^2^ = 0.23, F = 89.13, *p* < 0.01	-	-	-	-	-	-

Gender was dummy coded such that adolescent boy = 0 and adolescent girl = 1. Bootstrap sample size = 5000. LLCI = low limit confidence interval, ULCI = upper limit confidence interval, path a = the effect of maternal smartphone dependency on negative parenting, path c’ = the effect of maternal smartphone dependency on adolescent smartphone dependency, path b_1_ = the effect of negative parenting on adolescent smartphone dependency, path b_2_ = the effect of adolescent gender on adolescent smartphone dependency, path b_3_ = the conditional effect of adolescent gender on negative parenting to adolescent smartphone dependency. Values were controlled for covariates (perceived economic status, mother’s education, and adolescents’ depression and self-esteem).

**Table 3:** Conditional indirect effect of mother smartphone dependency on adolescent smartphone dependency through negative parenting by adolescent gender.

Gender	Indirect Effect	Boot SE	Boot LLCI	Boot ULCI
Boy	0.02	0.00	0.01	0.03
Girl	0.03	0.01	0.02	0.40

SE = standard error, LLCI = limit confidence interval, ULCI = upper limit confidence interval

## References

[1] Pew Research Center Smartphone Ownership Is Growing Rapidly around the World, But Not Always Equally. https://www.pewresearch.org/global/2019/02/05/smartphone-ownership-is-growing-rapidly-around-the-world-but-not-always-equally/.

[2] Ministry of Science and ICT (2020). Smartphone Overdependency Survey 2019.

[3] Duradoni M., Innocenti F., Guazzini A. (2020). Well-being and social media: A systematic review of Bergen addiction scales. Future Internet.

[4] Elhai J.D., Dvorak R.D., Levine J.C., Hall B.J. (2017). Problematic smartphone use: A conceptual overview and systematic review of relations with anxiety and depression psychopathology. J. Affect. Disord..

[5] Jun S. (2016). The reciprocal longitudinal relationships between mobile phone addiction and depressive symptoms among Korean adolescents. Comput. Hum. Behav..

[6] Kwak J.Y., Kim J.Y., Yoon Y.W. (2018). Effect of parental neglect on smartphone addiction in adolescents in South Korea. Child. Abuse Neglect..

[7] Llorca-Mestre A., Samper-García P., Malonda-Vidal E., Cortés-Tomás M. (2017). Parenting style and peer attachment as predictors of emotional instability in children. Soc. Behav. Personal..

[8] Lee H., Kim J.W., Choi T.Y. (2017). Risk factors for smartphone addiction in Korean adolescents: Smartphone use patterns. J. Korean Med. Sci..

[9] Pearson C., Hussain Z. (2016). Smartphone addiction and associated psychological factors. Turk. J. Addict..

[10] Goodman S.H., Gotlib I.H. (1999). Risk for psychopathology in the children of depressed mothers: A developmental model for understanding mechanisms of transmission. Psychol. Rev..

[11] De Rosnay M., Cooper P.J., Tsigaras N., Murray L. (2006). Transmission of social anxiety from mother to infant: An experimental study using a social referencing paradigm. Behav. Res. Ther..

[12] Guazzini A., Duradoni M., Capelli A., Meringolo P. (2019). An explorative model to assess individuals’ phubbing risk. Future Internet.

[13] Dowling N.A., Shandley K., Oldenhof E., Youssef G., Thomas S., Frydenberg E., Jackson A.C. (2016). The intergenerational transmission of problem gambling: The mediating role of parental psychopathology. J. Addict. Behav..

[14] Dowling N.A., Shandley K.A., Oldenhof E., Affleck J.M., Youssef G.J., Frydenberg E., Thomas S.A., Jackson A.C. (2017). The intergenerational transmission of at-risk/problem gambling: The moderating role of parenting practices. Am. J. Addict..

[15] Bandura A. (1977). Social Learning Theory.

[16] Xie X., Chen W., Zhu X., He D. (2019). Parents’ phubbing increases adolescents’ mobile phone addiction: Roles of parent-child attachment, deviant peers, and gender. Child. Youth Serv. Rev..

[17] Cho K.S., Lee J.M. (2017). Influence of smartphone addiction proneness of young children on problematic behaviors and emotional intelligence: Mediating self-assessment effects of parents using smartphones. Comput. Hum. Behav..

[18] Bae S.M., Choi Y.H., Song S.M., Cha S.E. (2017). Effects of mother’s smartphone dependency and maternal guilty feelings on early childhood emotion regulation. Korean J. Community Living Sci..

[19] Hur J., Ahn J.R. (2016). The effects of mothers’ smartphone addiction on parenting efficacy and young children’s social competence. Korea Inst. Child Care Educ..

[20] Kildare C.A., Middlemiss W. (2017). Impact of parents mobile device use on parent-child interaction: A literature review. Comput. Hum. Behav..

[21] Wolfers L.N., Kitzmann S., Sauer S., Sommer N. (2020). Phone use while parenting: An observational study to assess the association of maternal sensitivity and smartphone use in a playground setting. Comput. Hum. Behav..

[22] McDaniel B.T., Coyne S.M. (2016). Technology interference in the parenting of young children: Implications for mothers’ perceptions of coparenting. Soc. Sci. J..

[23] Jeong G.Y., Shin H.C. (2011). Validation of the Korean Version of Parents as Social Context Questionnaire (PSCQ). Korean J. Couns..

[24] Bae S. (2015). The relationships between perceived parenting style, learning motivation, friendship satisfaction, and the addictive use of smartphones with elementary school students of South Korea: Using multivariate latent growth modeling. Sch. Psychol. Int..

[25] Chiu S.I. (2014). The relationship between life stress and smartphone addiction on Taiwanese university student: A mediation model of learning self-efficacy and social self-efficacy. Comput. Hum. Behav..

[26] Lian L., You X., Huang J., Yang R. (2016). Who overuses smartphones? Roles of virtues and parenting style in smartphone addiction among Chinese college students. Comput. Hum. Behav..

[27] Liu F., Zhang Z., Chen L. (2020). Mediating effect of neuroticism and negative coping style in relation to childhood psychological maltreatment and smartphone addiction among college students in China. Child Abus. Negl..

[28] Yang Y.S., Yen J.Y., Ko C.H., Cheng C.P., Yen C.F. (2010). The association between problematic cellular phone use and risky behaviors and low self-esteem among Taiwanese adolescents. BMC Public Health.

[29] Lee J., Sung M.-J., Song S.-H., Lee Y.-M., Lee J.-J., Cho S.-M., Park M.-K., Shin Y.-M. (2018). Psychological factors associated with smartphone addiction in South Korean adolescents. J. Early Adolesc..

[30] Augner C., Hacker G.W. (2012). Associations between problematic mobile phone use and psychological parameters in young adults. Int. J. Public Health.

[31] Fattore L., Melis M., Fadda P., Fratta W. (2014). Sex differences in addictive disorders. Front. Neuroendocrinol..

[32] Ho R.C., Zhang M.W., Tsang T.Y., Toh A.H., Pan F., Lu Y., Cheng C., Yip P., Lam L., Lai C. (2014). The association between internet addiction and psychiatric co-morbidity: A meta-analysis. BMC Psychiatry.

[33] Levin I.P., Gaeth G.J. (1988). How consumers are affected by the framing of attribute information before and after consuming the product. J. Consum. Res..

[34] Maheswaran D., Meyers-Levy J. (1990). The influence of message framing and issue involvement. J. Mark. Res..

[35] Endendijk J.J., Groeneveld M.G., van der Pol L.D., van Berkel S.R., Hallers-Haalboom E.T., Bakermans-Kranenburg M.J., Mesman J. (2017). Gender differences in child aggression: Relations with gender-differentiated parenting and parents’ gender-role stereotypes. Child Dev..

[36] Perry N.B., Leerkes E.M., Dunbar A.S., Cavanaugh A.M. (2017). Gender and ethnic differences in young adults’ emotional reactions to parental punitive and minimizing emotion socialization practices. Emerg. Adulthood.

[37] Shin K., Kim D., Jung Y. (2011). Development of Korean Smart Phone Addiction Proneness Scale for Youth and Adults.

[38] Kim T., Lee E. (2017). Validation of the Korean version of parents as social context questionnaire for adolescents: PSCQ_KA. Korean J. Youth Stud..

[39] Skinner E., Johnson S., Snyder T. (2005). Six dimensions of parenting: A motivational model. Parent. Sci. Pract..

[40] Chu H.S., Tak Y.R., Lee H. (2020). Exploring psychosocial factors that influence smartphone dependency among Korean adolescents. PLoS ONE.

[41] Kim K.I., Kim J.H., Won H.T. (1984). Korean Manual of Symptom Checklist-90-Revision.

[42] Rosenberg M. (2015). Society and the Adolescent Self-Image.

[43] Hayes A.F. (2017). Introduction to Mediation, Moderation, and Conditional Process. Analysis: A Regression-Based Approach.

[44] Bai Q., Lei L., Hsueh F.H., Yu X., Hu H., Wang X., Wang P. (2020). Parent-adolescent congruence in phubbing and adolescents’ depressive symptoms: A moderated polynomial regression with response surface analyses. J. Affect. Disord..

[45] Liu Q.X., Fang X.Y., Deng L.Y., Zhang J.T. (2012). Parent–adolescent communication, parental Internet use and Internet-specific norms and pathological Internet use among Chinese adolescents. Comput. Hum. Behav..

[46] Vaala S.E., Bleakley A. (2015). Monitoring, mediating, and modeling: Parental influence on adolescent computer and Internet use in the United States. J. Child. Media.

[47] Bleakley A., Jordan A.B., Hennessy M. (2013). The relationship between parents’ and children’s television viewing. Pediatrics.

[48] Bogenschneider K., Wu M.Y., Raffaelli M., Tsay J. (1998). Parent influences on adolescent peer orientation and substance use: The interface of parenting practices and values. Child Dev..

[49] Hops H., Duncan T.E., Duncan S.C., Stoolmiller M. (1996). Parent substance use as a predictor of adolescent use: A six-year lagged analysis. Ann. Behav. Med..

[50] Coyne S.M., Holmgren H., Keenan-Kroff S., Petersen S., Stockdale L. (2020). Prenatal predictors of media use during infancy. Cyberpsychol. Behav. Soc. Netw..

[51] Bandura A. (1989). Human agency in social cognitive theory. Am. Psychol..

[52] Song S.M., Park B., Kim J.E., Park N.S. (2019). Examining the relationship between life satisfaction, smartphone addiction, and maternal parenting behavior: A south Korean example of mothers with infants. Child Indic. Res..

[53] Hyun E., Cho M.M., Cho K.S., Kim T.Y. (2013). A study of the relationships among mothers’ smartphone addiction levels, parenting efficacy and parenting stress. Korean J. Early Child. Educ..

[54] Chang Y.O. (2015). The effects of mothers’ smartphone addiction on parenting efficacy and parenting attitude. J. Korean Child Care Educ..

[55] Busch P.A., McCarthy S. (2020). Antecedents and consequences of problematic smartphone use: A systematic literature review of an emerging research area. Comput. Hum. Behav..

[56] Seo M., Choi E. (2018). Classes of trajectory in mobile phone dependency and the effects of negative parenting on them during early adolescence. Sch. Psychol. Int..

[57] Jahng K.E. (2019). Maternal abusive parenting and young South Korean adolescents’ problematic smartphone use: The moderating effects of time spent hanging out with peers and trusting peer relationships. Child. Youth Serv. Rev..

[58] Wang J.L., Wang H.Z., Gaskin J., Wang L.H. (2015). The role of stress and motivation in problematic smartphone use among college students. Comput. Hum. Behav..

[59] Choquet M., Hassler C., Morin D., Falissard B., Chau N. (2008). Perceived parenting styles and tobacco, alcohol and cannabis use among French adolescents: Gender and family structure differentials. Alcohol Alcohol..

[60] Lee E.J., Kim H.S. (2018). Gender differences in smartphone addiction behaviors associated with parent–child bonding, parent–child communication, and parental mediation among Korean elementary school students. J. Addict. Nurs..

[61] Ching K.H., Tak L.M. (2017). The structural model in parenting style, attachment style, self-regulation and self-esteem for smartphone addiction. IAFOR J. Psychol. Behav. Sci..

[62] Pardini D.A. (2008). Novel insights into longstanding theories of bidirectional parent–child influences: Introduction to the special section. J. Abnorm. Child Psychol..

[63] Koning I.M., Peeters M., Finkenauer C., Van Den Eijnden R.J. (2018). Bidirectional effects of Internet-specific parenting practices and compulsive social media and Internet game use. J. Behav. Addict..

[64] Leung J.T., Shek D.T. (2014). Parent–adolescent discrepancies in perceived parenting characteristics and adolescent developmental outcomes in poor Chinese families. J. Child Fam. Stud..

[65] Paulson S.E., Sputa C.L. (1996). Patterns of parenting during adolescence: Perceptions of adolescents and parents. Adolescence.

[66] Hwang Y., Choi I., Yum J.Y., Jeong S.H. (2017). Parental mediation regarding children’s smartphone use: Role of protection motivation and parenting style. Cyberpsychol. Behav. Soc. Netw..

[67] Collier K.M., Coyne S.M., Rasmussen E.E., Hawkins A.J., Padilla-Walker L.M., Erickson S.E., Memmott-Elison M. (2016). Does parental mediation of media influence child outcomes? A meta-analysis on media time, aggression, substance use, and sexual behavior. Dev. Psychol..

[68] McCart M.R., Priester P.E., Davies W.H., Azen R.J. (2006). Differential effectiveness of behavioral parent-training and cognitive-behavioral therapy for antisocial youth: A meta-analysis. J. Abnorm. Child Psychol..

[69] Forehand R., Lafko N., Parent J., Burt K.B. (2014). Is parenting the mediator of change in behavioral parent training for externalizing problems of youth?. Clin. Psychol. Rev..

